# Élaboration et évaluation de l’utilité, de l’utilisabilité et de l’acceptabilité de ressources éducatives produites en réponse à la crise de la COVID-19

**DOI:** 10.1177/1757975921996133

**Published:** 2021-03-18

**Authors:** Nicolas Tessier, Nathalie O’Callaghan, Carmella Fernandez Da Rocha Puleoto, Didier Jourdan

**Affiliations:** 1 Chaire UNESCO et centre collaborateur OMS « ÉducationS & Santé », Université Clermont-Auvergne, Nouméa, Nouvelle-Calédonie; 2 DENC (Direction de l’enseignement de la Nouvelle-Calédonie)

**Keywords:** promotion de la santé, COVID-19, continuité pédagogique, participation, utilité, utilisabilité, enseignement primaire

## Abstract

L’éducation est l’un des principaux déterminants de la santé. Or, la crise sanitaire a conduit à ce que 90% de la population étudiante mondiale, soit 1,57 milliard d’enfants et de jeunes dans 190 pays, soient privés d’école. Les conséquences de la fermeture des écoles sur les apprentissages et la santé sont bien établies. L’impact du confinement sur la santé des enfants et des jeunes est plus marqué que celui sur d’autres groupes d’âge car l’interaction entre pairs est un aspect essentiel du développement. De plus, les conséquences de la fermeture des écoles sont d’autant plus importantes que les élèves sont plus vulnérables socialement. Le dispositif de promotion de la santé « Réussir, être bien, être ensemble » est une démarche de co-contruction d’outils pédagogiques en éducation à la santé qui prend en compte la diversité culturelle, s’appuie sur les pratiques existantes, les partage et les enrichit des apports de la recherche (processus de conception continuée dans l’usage). Il a été activé pour élaborer avec les acteurs, et dans un temps très court, un ensemble d’outils à destination des écoles primaires en vue de s’assurer de la continuité pédagogique pendant la crise. L’évaluation de ces outils auprès des professionnels en activité et en formation (n = 50) montre qu’ils ont une bonne utilisabilité en référence aux pratiques de classe existantes (score de 8,2 sur 10) et aux besoins des élèves (score de 8 sur 10), une utilité pour le développement de compétences et de connaissances en éducation à la santé (score de 8,4 sur 10), une acceptabilité par rapport aux approches pédagogiques, aux supports contextualisés et à leur mise en œuvre (score de 8,3 sur 10). Cette étude montre que la promotion de la santé comme approche est susceptible d’offrir un cadre pour l’élaboration d’outils d’intervention adaptés en période de crise sanitaire.

## Introduction

Agir sur les milieux de vie et développer les compétences de chacun afin de créer les conditions, pour les personnes et les communautés, de prendre en charge leur santé, telle est la finalité de la promotion de la santé (1). Comme discipline au sein de la santé publique et comme champ de pratiques, la promotion de la santé est appelée à contribuer à la mobilisation collective face à la crise de la COVID-19. Comme le souligne Stephan Van den Broucke, elle intervient en aval, en mettant l’accent sur le changement de comportement individuel et la littératie en santé, au niveau intermédiaire via des interventions touchant les organisations et les communautés, et en amont en informant les politiques touchant la population (2).

Cet article explore les potentialités de la mise en œuvre d’un dispositif de promotion de la santé en milieu scolaire visant à limiter les conséquences de la fermeture des écoles sur les élèves, en particulier les plus vulnérables d’entre eux. En effet, l’éducation est l’un des principaux déterminants de la santé. Or, la crise sanitaire a conduit à ce que 90 % de la population étudiante mondiale, soit 1,57 milliard d’enfants et de jeunes dans 190 pays, soient privés d’école (3). Les conséquences de la fermeture des écoles sur les apprentissages (4) et la santé (5) sont bien établies. L’impact du confinement sur la santé des enfants et des jeunes est plus marqué que celui sur d’autres groupes d’âge car l’interaction entre pairs est un aspect essentiel du développement. De plus, l’effet de la fermeture des écoles est d’autant plus important que les élèves sont plus vulnérables socialement (6) ou du fait de problèmes de santé (7). Des enquêtes menées auprès des enseignants ont montré que les élèves défavorisés ont appris de 25 à 50 % de moins qu’ils ne l’auraient fait en classe. La même étude montre que l’écart de réussite entre les élèves favorisés et les élèves défavorisés a triplé pendant le confinement (8).

Créer les conditions de l’apprentissage et de la socialisation à distance, renforcer le lien école-famille pendant le confinement apparaissent ainsi comme des enjeux majeurs (9).

Le dispositif de promotion de la santé « Réussir, être bien, être ensemble » (REBEE) développé en Nouvelle-Calédonie s’appuie sur la participation des acteurs de terrain de la santé et de l’éducation, un partage des savoirs entre ces différents partenaires et la recherche, un pilotage local et réactif sur la base d’une organisation fortement soutenue par le gouvernement calédonien (10). Il s’agit d’une démarche de formation, d’accompagnement et de co-contruction d’outils pédagogiques en éducation à la santé qui prend en compte la diversité culturelle, s’appuie sur les pratiques existantes, les partage et les enrichit des apports de la recherche (processus de conception continuée dans l’usage). Il a été activé pour co-élaborer avec les acteurs, et dans un temps très court, un ensemble d’outils à destination des écoles primaires en vue de s’assurer de la continuité pédagogique. Ces outils ont été partagés non seulement avec les enseignants et les parents, mais également avec les acteurs de santé à l’échelle des communautés de façon à soutenir leur utilisation sur le terrain.

Cet article vise à décrire les modalités de développement des outils d’accompagnement des écoles pendant la crise de la COVID-19 et à présenter les résultats de l’évaluation préliminaire dont ils ont été l’objet. Destinés à l’accompagnement des professionnels en exercice, mais également à la formation des futurs professionnels, ces outils ont été présentés à des enseignants sur le terrain et à des étudiants.

Après avoir présenté le contexte spécifique de la Nouvelle-Calédonie, puis les caractéristiques du dispositif de promotion de la santé REBEE, nous détaillerons les modalités d’élaboration des outils pédagogiques et décrirons les résultats de l’évaluation de l’utilité, de l’utilisabilité et de l’acceptabilité des outils.

## Contexte

### Le contexte néo-calédonien

Notre étude s’est déployée en Nouvelle-Calédonie, archipel français du Pacifique voisin de l’Australie et de la Nouvelle-Zélande. Sa population est de 282 200 habitants (11). Politiquement, le pays dispose d’une grande autonomie suite aux Accords de Nouméa et une répartition des compétences est mise en œuvre entre les différentes institutions du territoire (12). Très diversifié sur le plan culturel et des modes de vie sociétaux, l’archipel se caractérise par un très haut niveau d’inégalités avec, par exemple, une espérance de vie supérieure de 4 ans en province Sud par rapport aux îles Loyauté (13).

En 2016, les élus du Congrès ont adopté la Charte d’application des orientations de politique éducative de la Nouvelle-Calédonie. Cette Charte comprend un plan d’action visant à atteindre les quatre ambitions fixées par le projet éducatif de la Nouvelle-Calédonie : développer l’identité de l’école calédonienne, considérer la diversité des publics pour une école de la réussite pour tous, ancrer l’école dans son environnement pour un climat scolaire au service de l’épanouissement de l’élève, et ouvrir l’école sur la région Océanie et sur le monde (14). Fin 2018, le pays s’est doté d’un plan de santé intitulé « Do Kamo, Être épanoui » qui vise à réformer le modèle économique et la gouvernance du système de protection sociale et de santé pour la période 2018 - 2028. Ce plan s’appuie sur la prise en compte des déterminants de la santé des populations et vise l’amélioration de la santé de tous et la réduction des inégalités de santé (15,16). Ces deux textes de référence constituent les éléments clés sur lesquels s’appuie le dispositif REBEE.

### Les enseignants et la promotion de la santé à l’école primaire

Les études réalisées dans l’enseignement primaire montrent que, selon les contextes, les enseignants s’emparent différemment des problématiques de santé. Dans beaucoup de cas, ils rencontrent des difficultés (17). Ils n’accordent habituellement qu’une faible priorité à la promotion de la santé et n’ont pas toujours conscience de leur rôle en éducation à la santé (18). De multiples facteurs conditionnent à la fois le sentiment pour les enseignants d’être légitimes à éduquer à la santé et leurs pratiques dans ce domaine (19).

Selon Bryk (20), le meilleur moyen pour faire évoluer les pratiques professionnelles est d’amorcer une collaboration chercheur/enseignant le plus tôt possible dans la conception d’un outil pour connaître et mieux intégrer les habitudes professionnelles des enseignants. En effet, l’intégration de pratiques innovantes dépendraient de deux facteurs principaux : la compatibilité avec les pratiques habituelles des professeurs et l’efficience de l’intervention, c’est-à-dire du rapport entre son coût pour les enseignants et ses bénéfices (21). Des études montrent que les outils doivent (22, 23) :

- répondre aux préoccupations des enseignants et aux besoins des élèves ;
- s’intégrer sans trop de bouleversements dans les conditions d’exercice des enseignants ;
- inclure des justifications théoriques et empiriques pour en comprendre les principes.

Sur la base de ces données, le dispositif REBEE accorde ainsi une place centrale à la conception, à la formation et à l’accompagnement de l’utilisation d’outils professionnels.

### Le dispositif « Réussir, être bien, être ensemble »

REBEE est un dispositif de promotion de la santé qui vise spécifiquement la co-construction d’outils pédagogiques en éducation à la santé pour l’école primaire (24). En se basant sur les travaux relatifs à la conception continuée dans l’usage, il a été lancé en 2018. Il est ancré dans une démarche de promotion de la santé à l’école (25) et est structuré autour de trois grands axes : le rapport à soi, le rapport aux autres et le rapport à l’environnement.

Ce travail a conduit à la co-production de fiches détaillées et opérationnelles destinées à tous les enseignants afin qu’ils puissent soit les utiliser en l’état, soit les adapter à leur projet de classe. Pour cela, des scenarii pédagogiques aux ressources diverses, ancrages disciplinaires multiples et aux approches pédagogiques variées sont proposés (10).

Ce dispositif s’appuie sur un pilotage partagé (direction de l’éducation et agence sanitaire) qui rend possible la participation de tous les partenaires décisionnels et acteurs du terrain. L’ensemble génère un partage des savoirs et des pratiques. Les acteurs du secteur de la santé, de par leur expertise, procurent un appui scientifique, un soutien opérationnel et des ressources pour le monde de l’éducation (2,26). L’implication du monde de l’éducation favorise les chances de réussite d’une innovation pédagogique ou de la diffusion d’un outil.

La mise en œuvre du dispositif s’est organisée en trois temps successifs.

Le premier temps (octobre 2018 à mai 2019) était la co-conception d’un premier prototype par un groupe constitué d’enseignants, de cadres de l’éducation et de professionnels de santé.

Le second temps (avril 2019 à septembre 2019) correspondait à la phase de mise en œuvre des activités du prototype dans 46 écoles de Nouvelle-Calédonie et à la formation des enseignants. Les enseignants disposaient d’éléments pour expérimenter l’outil, faire leurs propositions de séances et partager leurs retours sur celles du prototype. Pendant cette phase, ils étaient accompagnés à l’échelon local par 14 directeurs d’écoles et 3 conseillers pédagogiques.

La troisième étape (septembre 2020) prévoit la diffusion des différents outils, l’évaluation de son impact sur les compétences des élèves et l’amélioration de l’outil sur la base des contributions d’un plus grand nombre d’enseignants.

La crise sanitaire a conduit à accélérer la diffusion des fiches pour assurer la continuité pédagogique.

## Le développement des outils de continuité pédagogique

En Nouvelle-Calédonie, la période de confinement total sur l’ensemble du territoire a débuté le 25 mars 2020. Le travail sur les outils de continuité pédagogique en éducation à la santé a commencé la semaine précédente, dès l’annonce du confinement. Nous avons pris en compte deux éléments qui nous apparaissaient cruciaux : les représentations des enseignants sur l’éducation à la santé et les compétences des élèves devant être développées en période de COVID-19. Nous nous sommes appuyés sur une étude qualitative réalisée en février 2020 : nous avions interrogé en présentiel via un entretien semi-guidé 17 enseignants répartis sur l’ensemble du territoire et exerçant dans différents milieux (urbain aisé, urbain défavorisé, urbain mixte, village, tribu) afin d’identifier leurs représentations de l’éducation à la santé et leurs pratiques de classe. Leurs réponses ont orienté la définition des contenus proposés lors de la continuité pédagogique. Nous avons ensuite mobilisé les acteurs du dispositif REBEE. Un travail collectif a été engagé de façon à identifier, parmi les ressources déjà élaborées dans le cadre de ce dispositif, des supports permettant aux enseignants et aux familles d’aborder les problématiques suivantes en période de pandémie et de confinement : éviter la propagation des virus, gérer ses émotions et son bien-être, construire un esprit critique face aux informations. D’autres ressources ont été identifiées et mises à disposition. Un site internet (27) a été créé et est accessible via le site Internet de la Direction de l’Enseignement de la Nouvelle-Calédonie (DENC). Ce site dédié regroupe 36 fiches destinées aux éducateurs des enfants entre 6 et 11 ans (parents, enseignants et professionnels de la santé intervenant dans les écoles) avec pour objectif de leur permettre d’aborder les thématiques de santé suivantes : microbes et gestes d’hygiène, bien-être et connaissance de soi, médias et information, santé et attitude. En ce qui concerne les parents, c’est l’enseignant, dans le cadre de sa progression, qui les invite à travailler telle ou telle fiche à la maison. Différentes solutions ont été expérimentées pour permettre aux gens sans connexion Internet d’accéder aux ressources : permanence dans les écoles, distribution des activités sous format papier ou sur clé USB dans le respect des gestes barrières par les enseignants, ou les gendarmes pour les lieux isolés.

Toutes les fiches proposées étaient construites selon un modèle identique afin de favoriser leur utilisation. Le recto, destiné à la classe, aux élèves, supportant essentiellement un ou plusieurs documents iconographiques (photo, dessin, reproduction d’affiches, texte). Le verso, destiné à l’enseignant et aux parents, présente la trame de l’exploitation pédagogique du document iconographique. En fonction de l’intention pédagogique, chaque fiche pouvait être téléchargée indépendamment. Ces ressources sont complétées par des vidéos de personnels de la santé et de l’éducation qui proposent aux enseignants et aux familles des éléments sur leur utilisation.

Fin juin 2020 le site avait accueilli 1900 visiteurs différents pour 16 000 pages consultées. Les fiches les plus téléchargées sont celles relatives aux microbes, au lavage des mains et à l’eau. Les visites d’une durée supérieure à 30 minutes représentent 17,3 %, 31,5 % et 44,8 % de l’ensemble en avril, mai et juin.

## Évaluation de l’utilité, de l’utilisabilité et de l’acceptabilité des outils

Dans le but de s’assurer de la pertinence des ressources proposées, un dispositif d’évaluation a été élaboré. Cet article rend compte de la première phase de l’évaluation centrée sur l’utilité, l’utilisabilité et l’acceptabilité des outils. Une évaluation à long terme incluant l’impact sur les compétences des enfants est également en cours, ses résultats seront disponibles courant 2021.

### Le questionnaire

Les outils étant destinés à la fois à des professionnels en activité et à des étudiants en formation initiale, cette évaluation a été réalisée avec des enseignants calédoniens non impliqués dans le dispositif de co-conception ainsi qu’avec des étudiants en promotion de la santé.

Cette étude qualitative est basée sur un questionnaire administré en ligne. Trente enseignants des trois provinces ont été invités par email à participer à cette évaluation. Ces enseignants ont été contactés à notre demande par les équipes de circonscriptions de l’archipel et ne constituent pas un échantillon représentatif. Le questionnaire incluait 9 questions et a été ouvert entre le 10 avril 2020 et le 27 mai 2020. Les questions portaient sur : l’adaptabilité des fiches, l’attractivité des activités, la pertinence des scénarios pédagogiques, la nécessité d’une formation pour utiliser ces fiches, les points positifs et les points à améliorer. Pour chaque question, les enseignants se positionnaient sur une échelle de 1 à 10 et explicitaient leur positionnement par un commentaire. Cette approche mixte selon Creswell (28) permet de disposer de données qualitatives et quantitatives fournissant différents types d’informations pour prendre en compte la complexité des dispositifs (29) malgré un faible échantillon.

S’appuyant sur les travaux de Tricot (30,31) et de Goigoux (32), ce questionnaire vise à mesurer en quoi les outils répondent aux attentes des professionnels en matière de santé, en période de crise sanitaire. Les données recueillies ont été catégorisées, saisies sur des tableurs et codées pour être analysées de façon quantitative selon 3 critères différents mais complémentaires : l’utilité, l’utilisabilité et l’acceptabilité.

L’utilité a pour but de définir l’efficacité pédagogique de l’outil. Autrement dit de savoir si les activités proposées dans l’outil permettent d’atteindre l’objectif visé et si elles sont pertinentes en termes de motivation et d’apprentissages pour les élèves.

L’utilisabilité mesure la possibilité offerte à l’enseignant d’utiliser, de réutiliser l’outil dans l’état actuel, voire de le modifier ou de l’adapter à ses pratiques et/ou ses élèves.

L’acceptabilité concerne la décision d’utiliser ou non l’outil proposé. Il s’agit de mesurer si l’outil répond aux prescriptions institutionnelles, son intérêt, et la compatibilité avec ses représentations du métier en termes de styles et démarches pédagogiques.

## Résultats

Cinquante personnes ont rempli le questionnaire (30 enseignants calédoniens et 20 étudiants de master première année en santé publique).

### Le point de vue des enseignants

Les enseignants interrogés sont expérimentés : 80 % enseignent depuis plus de 10 ans et 20 % ont entre 5 et 10 ans d’ancienneté. Ils exercent en moyenne depuis 6,5 ans dans leur niveau d’enseignement actuel au sein de différents cycles et niveaux de l’école primaire : trois enseignants en maternelle (enfants de 3 à 6 ans), 13 enseignants en cycle 2 (enfants de 6 à 9 ans), 17 enseignants sont au cycle 3 (enfants de 9 à 11 ans) et deux enseignants exercent sur plusieurs cycles à la fois.

L’utilisabilité de l’outil est mise en avant par les enseignants. Ils déclarent que les fiches sont adaptées ou facilement adaptables à leurs pratiques de classe (score de 8,2 sur 10) ainsi qu’à leurs élèves (score de 8 sur 10). L’adaptabilité des fiches aux pratiques et aux élèves est souvent mise en avant :*Les documents sont suffisamment ouverts et conçus pour le cycle, ce qui nous laisse une marge d’adaptation. Ces documents sont accessibles tout en conservant une certaine résistance pour prolonger la réflexion.Le niveau de connaissances et de réflexion de mes élèves leur permettrait de participer activement à ses activités et ils seraient intéressés par les thèmes proposés. Certaines activités sont toutefois un peu difficiles pour mes élèves.*

Par ailleurs, la question autour de la nécessité d’être formé pour utiliser cet outil montre que la plupart des enseignants n’en n’éprouvent pas le besoin (4,2 sur 10). En effet, les symboles de chaque étape, leur concision en font un outil intuitif et facilement utilisable selon leurs dires ( « Le déroulement des séances est très détaillé, les notions abordées sont simples et des pistes sont données aux enseignants pour se documenter. »). À noter que six enseignants mentionnent la nécessité d’une formation dans le but de favoriser une plus large diffusion de l’outil et de permettre de mieux comprendre les enjeux de l’éducation à la santé.

L’utilité des fiches est mentionnée par les enseignants pour ce qui concerne le développement de compétences et de connaissances en éducation à la santé pour leurs élèves (score de 8,4 sur 10). Les enseignants mentionnant par ailleurs que ces notions sont très peu abordées à l’école ( « Permet d’aborder des notions souvent mises de côté dans les apprentissages car vu comme acquises au sein des familles comme le lavage des mains, le temps devant les écrans. »). Ces fiches pourront leur permettre également de mettre en relation les activités qu’ils mènent déjà avec les questions d’éducation à la santé. Le but étant alors de les utiliser pour approfondir certaines notions et participer ainsi de manière importante à la construction de compétences en santé.

L’aspect motivationnel leur est apparu aussi comme un vecteur important pour permettre à leurs élèves de rentrer dans les activités (score de 8,3 sur 10). Ces fiches sont jugées comme intéressantes et motivantes de par la variété des supports et la contextualisation de l’apprentissage suscitant chez les élèves curiosité et questionnement.

Enfin, l’acceptabilité de l’outil semble bonne, en particulier sur les aspects suivants :

- L’approche ludique : *« Elles abordent de façon ludique les notions en permettant aux élèves d’échanger leur point de vue et d’apprendre ensemble. » ; « Les documents proposés à l’étude sont pertinents, les activités ludiques, la construction des fiches est claire et fonctionnelle, je trouve que c’est un très bel outil. » ;*
- Une complémentarité avec les pratiques existantes due à la variété des supports et des situations : *« La pluralité des supports et les débats en fin de séances très intéressantes. » ; « Ces fiches peuvent venir en complément de ce qui est déjà mis en place dans la classe ou pour des notions qui pourraient être abordées. » ;*
- La construction des fiches : *« Ces fiches comportent plusieurs phases qui correspondent à ma pratique : un retour au calme et à la concentration, une phase de débat, des lectures ou vidéos documentaires qui pourraient être transformées en cartes mentales. » ; « Le déroulement "découverte/activité/pour aller plus loin" similaire à celui pratiqué en classe. » ;*
- La contextualisation de l’outil : *« Les activités sont contextualisées à la Calédonie et l’enfant est au centre des questions ou des activités proposées (on lui demande d’agir, de donner son avis.). »*

### Le point de vue des étudiants en santé

L’utilisabilité du site et des fiches est mise en avant dans les différents retours en termes de facilité, de confort, de flexibilité et d’ajustement. En effet, les fiches proposées sur le site internet sont jugées facilement utilisables ou adaptables par le monde de la santé. L’organisation thématique du site et la description de l’ensemble des activités et des supports proposés (images, vidéos) sont très appréciées.

Le contenu des activités semble adapté aux élèves et faisable en famille dans le contexte sanitaire de la COVID-19.

En ce qui concerne l’utilité, les compétences et les connaissances ciblées en éducation à la santé dans le contexte de la COVID-19 ainsi que le choix des supports d’apprentissage sont jugés très pertinentes et en lien avec les déterminants de santé ciblés.

Par rapport à d’autres outils utilisés par le monde de la santé, des spécificités de l’outil ont également mis en avant notamment l’adaptation des fiches au contexte, la présence d’activités complémentaires permettant d’approfondir la notion, la présence d’un déroulement détaillé se rapprochant du monde de l’enseignement.

Enfin, en termes d’acceptabilité, les fiches sont majoritairement compatibles avec la vision de l’éducation à la santé qu’ont les étudiants, particulièrement en ce qui concerne le développement des compétences psychosociales. Le traitement des différents aspects de l’éducation à la santé comme étant une vision globale et pas uniquement hygiéniste est aussi un élément mis en avant.

**Figure 1.**
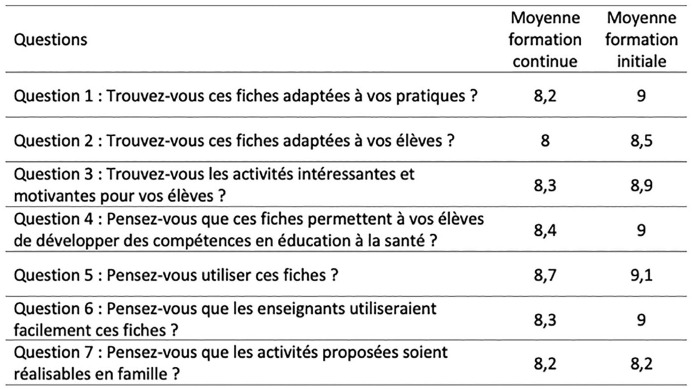
Moyenne des scores attribués pour chacune des questions par les enseignants en poste (formation continue) et les étudiants (formation initiale) pour l’utilité, l’utilisabilité et l’acceptabilité des outils.

La figure 1 récapitule les données relatives à l’utilité, à l’utilisabilité et à l’acceptabilité des outils.

## Discussion

Dans le contexte de la pandémie, nous avons développé un ensemble d’outils destinés à soutenir les parents, les enseignants et les professionnels de santé travaillant au sein des communautés. L’évaluation de l’utilité, de l’utilisabilité et de l’acceptabilité des outils tant par des enseignants expérimentés que par des professionnels en formation montre que ceux-ci sont perçus comme pertinents. Cependant, la faible taille de l’échantillon nous amène à considérer les résultats comme indicatifs. De plus, cette première évaluation ne prend pas en compte l’impact sur les enfants ni le taux d’implantation de l’outil par les enseignants. Cela fera l’objet d’une autre étude dont les résultats seront disponibles courtant 2021.

Les enseignants non impliqués dans ce processus de co-construction de cet outil ont accueilli de manière positive un outil « clé en main ». Il est perçu comme utile pour aborder l’éducation à la santé ; utilisable car simple et clair ; acceptable car en lien avec les pratiques existantes et contextualisé aux problématiques de leurs classes. Cet accueil positif de l’outil peut donc permettre de penser que les enseignants pourront mieux aborder l’éducation à la santé en classe et en comprendre les enjeux pour leurs élèves, notamment en ce qui concerne la réduction des inégalités. Même si à l’heure actuelle nous ne disposons pas d’éléments pour évaluer l’impact de l’outil REBEE sur ces inégalités, son accueil favorable par les enseignants dans les différentes provinces de l’archipel, notamment dans les tribus les plus éloignées des centres urbains, apparaît comme un indicateur de sa cohérence culturelle et pédagogique au sein des différents espaces de Nouvelle-Calédonie. Il a donc le potentiel de contribuer au développement de connaissances et de compétences des élèves les plus vulnérables.

Il est possible d’identifier les conditions du succès d’une telle entreprise. Il s’agit d’abord du soutien politique à une telle approche intersectorielle et une gouvernance adaptée. Dans le cas du dispositif REBEE-COVID-19 :

- un soutien politique via le plan Do Kamo adopté en 2016 à l’unanimité par le congrès de la Nouvelle-Calédonie ;
- un pilotage local et réactif, réunissant la chaire UNESCO « ÉducationS & Santé » et des cadres locaux de l’éducation et de la santé ;
- une participation des acteurs de terrain de la santé et de l’éducation. Dans le contexte de la promotion de la santé, les acteurs de la santé, de par leur expertise, procurent un appui scientifique et des ressources pour le monde de l’éducation ;
- un partage des savoirs entre ces différents partenaires et la recherche.

Par ailleurs, la mise à disposition d’un outil co-conçu par la santé et l’éducation a permis de consolider le rôle de l’école dans la promotion de la santé en période de crise sanitaire. Sans l’outil REBEE-COVID-19, un message uniquement sanitaire aurait été porté par l’ensemble des institutions sanitaires et sociales du pays.

Des difficultés ont néanmoins été observées. Il s’agit tout d’abord de l’injonction hiérarchique qui a communiqué principalement en début de confinement autour de la mise en place d’activités autour du français et des mathématiques. La question de l’éducation à la santé étant alors jugée secondaire à différents niveaux alors que de nombreuses recherches évoquées précédemment ont montré un lien fort entre la santé et la réussite scolaire. Il s’agit ensuite de la difficulté à contacter les parents pendant le confinement pour diverses raisons (absence de connexion ou de matériel numérique, numéros ou adresses email non valides, impossibilité à réaliser les tâches demandées car trop complexes ou non adaptées aux élèves sans la présence de l’enseignant), ce qui n’a pas toujours permis aux enseignants de pouvoir élargir cette continuité pédagogique.

Il apparaît que la démarche conduite dans un cadre temporel très contraint a bénéficié de son insertion dans une dynamique partenariale associant l’ensemble des acteurs autour du dispositif REBEE. Le fait de pouvoir s’appuyer sur une démarche en cours de promotion de la santé a permis de produire des outils adéquats et compatibles avec les différents contextes culturels et pratiques pédagogiques en Nouvelle-Calédonie. Visée émancipatrice, ancrage dans une stratégie visant les différents déterminants de la santé, sensibilité aux enjeux culturels et territoriaux, appui et valorisation des pratiques existantes, prise en compte des données de la recherche, partage des savoirs et participation sont les constituants de l’approche mise en place.
